# Prediction of Appropriate Prosthesis Length Using Preoperative Computed Tomography in Stapes Surgery With Transcanal Endoscopic Ear Surgery

**DOI:** 10.7759/cureus.100615

**Published:** 2026-01-02

**Authors:** Chiaki Nakahama, Yasuyuki Kajimoto, Yuki Koda, Makoto Moriguchi, Kishiko Sunami

**Affiliations:** 1 Otolaryngology, Osaka Metropolitan University, Osaka, JPN; 2 Otolaryngology, Moriguchi ENT Clinic, Osaka, JPN

**Keywords:** computed tomography, endoscopic surgery, prosthesis, stapes surgery, transcanal endoscopic ear surgery

## Abstract

Background

Transcanal endoscopic ear surgery (TEES) is becoming increasingly popular in otologic surgery and can be considered the first choice for stapes surgery. In this study, we analyzed a case series of TEES for stapes surgery, focusing on the estimation of appropriate prosthesis length using preoperative CT based on postoperative hearing results.

Methods

We retrospectively examined 31 patients who underwent TEES using a rigid endoscope for stapes surgeries. Postoperative hearing results were good in 27 cases (87.1%) and poor in four cases (12.9%). We reviewed the preoperative CT scans of all the patients and measured the straight-line distance from the incudostapedial joint to the oval window in the coronal images, assuming it to be the estimated appropriate prosthesis length (eAPL).

Results

The actual length of the prosthesis inserted during the operation was shorter than the eAPL in three of the four cases without postoperative hearing improvement. In the remaining patients, postoperative CT indicated that the prosthesis position was inadequate.

Conclusion

The measurement of the APL using preoperative CT could contribute to improving postoperative hearing outcomes after stapes surgery using TEES.

## Introduction

Transcanal endoscopic ear surgery (TEES) is becoming more popular in otologic surgery because of its advantages over conventional microscopic ear surgery [1-3], including a clear, magnified field of view with fewer blind spots; no need for a posterior incision in the ear; and less postoperative pain due to less bone removal. However, TEES has the disadvantages of requiring a one-handed surgical operation and the difficulty of grasping the three-dimensional position of the prosthesis inserted due to the two-dimensional observation of the surgical field. Thus, indications for TEES, particularly in stapes surgery, must be carefully considered. Although recent studies have demonstrated the feasibility and safety of endoscopic stapes surgery, with outcomes comparable to those of microscopic techniques, there is still a substantial gap in the literature regarding the role of preoperative imaging in surgical planning. In current standard practice, prosthesis length is determined intraoperatively by directly measuring the distance between the long process of the incus and the stapes footplate using a dedicated depth gauge, with an additional length of approximately 0.25 mm added to allow appropriate insertion of the prosthesis into the vestibule [4,5].

In particular, few studies have investigated whether high-resolution temporal bone CT can aid in predicting the ideal piston length for prosthesis placement, a factor critical to optimizing postoperative hearing outcomes [1]. Addressing this knowledge gap could enhance the precision and success of TEES, particularly in cases where suboptimal piston length contributes to poor audiological results. This study aimed to evaluate whether a simple preoperative CT-based measurement correlates with surgical outcomes and could serve as a reliable predictor of optimal prosthesis length.

## Materials and methods

This study included 31 patients (31 ears) who underwent endoscopic stapes surgery using a rigid endoscope (Olympus, Tokyo, Japan) at the Department of Otolaryngology, Osaka City University Hospital, and Osaka Metropolitan University between January 1, 2017, and April 30, 2021. All surgeries were performed by the same surgeon, and Fisch's reversal step stapedotomy [6] was performed in all cases. The following features were analyzed: hearing changes before and after surgery, complications related to surgery, the relationship between the linear distance from the oval window to the long process of the incus measured using preoperative CT, the length of the intraoperatively-inserted prosthesis (Olympus, Tokyo, Japan), and postoperative changes in hearing acuity. The final prosthesis length was determined intraoperatively using a dedicated depth gauge. Preoperative CT measurements were performed for descriptive comparison and were not used to guide prosthesis selection.

This study was conducted as a retrospective review and was approved by the institutional review board. The requirement for informed consent was waived due to the retrospective nature of the analysis.

Inclusion criteria were patients who underwent endoscopic stapes surgery, had a diagnosis of otosclerosis or ossicular malformations, had primary surgery, and had complete preoperative evaluations, including pure-tone audiometry and CT.

Preoperative audiometry was performed the day before surgery, and postoperative audiometry was conducted approximately one year after surgery, once the surgical site had stabilized. Hearing outcomes were classified as improved, unchanged, or worsened according to the criteria of the Japan Otological Society [7]. Temporal bone CT was performed at the time of diagnosis and again when surgery was scheduled.

**Figure 1:**
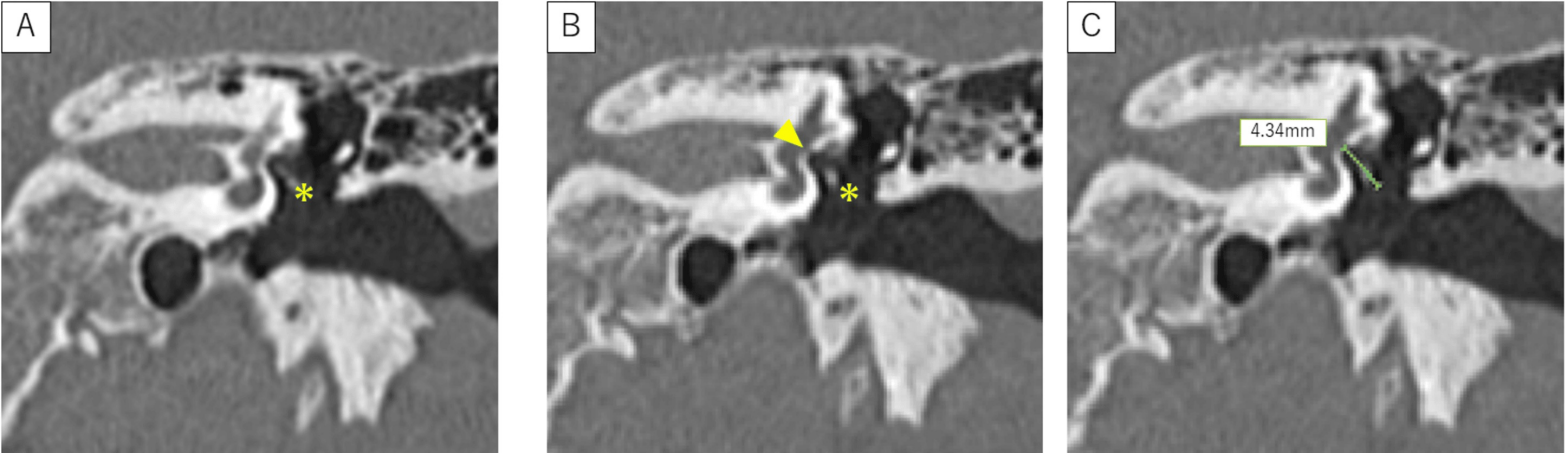
Estimation of the appropriate prosthesis length (eAPL) using thin-slice CT of the left temporal bone (coronal view) A: Identification of the incudostapedial joint on thin-slice CT images obtained under bone window settings. The cursor was fixed at the distal end of the long process of the incus (asterisk). B: Identification of the oval window on an adjacent slice located a few sections away. For coronal measurements, the oval window is defined on the slice in which the stapes footplate was most clearly visualized adjacent to the vestibule and inferior to the facial nerve canal. The straight-line distance from the fixed cursor to the oval window was measured (arrowheads). C: The linear distance between the cursor position in (A) and the oval window represents the estimated appropriate prosthesis length (eAPL).

The measurement method consisted of three steps (see Figure 1). In the first step, a temporal bone CT was obtained with a slice thickness of 0.6 mm and a reconstruction interval of 0.3 mm under bone window settings. Using thin-slice images, the incudostapedial joint was identified, and the cursor was fixed at a position corresponding to the distal end of the long process of the incus. All measurements were performed manually using the built-in caliper tool of the Picture Archiving and Communication System (PACS) workstation, without the use of dedicated three-dimensional reconstruction software.

In the second stop, the oval window was identified on adjacent slices shifted by a few sections from the incudostapedial joint. For coronal measurements, it was defined on the slice where the stapes footplate was most clearly visualized adjacent to the vestibule and inferior to the facial nerve canal (see Figure 1).

In the third step, the straight-line distance from the cursor to the oval window was measured. This distance was assumed to represent an estimate of the appropriate prosthesis length (eAPL). These measurements were performed on both coronal and axial CT images using the same methodology. For clarity, Figure 1 illustrates the measurement procedure on a representative coronal CT image.

Hearing outcomes were evaluated according to the Guidelines for Reporting Hearing Results in Middle Ear and Mastoid Surgery (2010) of the Japan Otological Society [7], using the four-frequency average. Postoperative success was defined as meeting at least one of the following criteria: (1) air-bone gap ≤15 dB, (2) hearing improvement ≥15 dB, or (3) postoperative hearing level ≤30 dB.

Complications were defined as new symptoms not present preoperatively, including vertigo, tinnitus, sensorineural hearing loss, or obvious intraoperative perilymphatic leakage.

Comparative analyses were performed to evaluate changes in hearing levels based on the criteria of the Japan Otological Society [7]. The relationship between eAPL and the length of the inserted prosthesis was assessed by comparing their numerical values. A p-value was not applied because no statistical tests were conducted for these analyses.

## Results

The baseline characteristics of the patients are summarized in Table 1. Of the 31 patients, 15 (48.4%) were male, and 16 (51.6%) were female; 11 cases involved the right ear and 20 involved the left ear. There were no cases of simultaneous left or right surgeries. There were five duplicate cases, one of which was a contralateral operation, and four were ipsilateral re-operations. Postoperative hearing was improved in 27 patients (87.1%) and not improved in four patients (12.9%). There were no cases of postoperative worsening of the sensorineural hearing loss. The diseases included 24 cases (77.4%) of otosclerosis and seven cases (22.6%) of ossicular malformations, including stapes fixation, one of which was complicated by incudostapedial joint separation. Twenty-two (71.0%) of the surgically-inserted prostheses were teflon wire pistons, and nine (29.0%) were teflon pistons (Table 1).

**Table 1:** Clinical characteristics of 31 patients

Clinical characteristics		n (%)
Sex	Men	15 (48.4%)
	Women	16 (51.6%)
Age, median (range)		47 years (17-68)
Side	Right	11 (35.5%)
	Left	20 (64.5%)
Disease	Otosclerosis	24 (77.4%)
	Ossicular malformation	7 (22.6%)
Postoperative hearing	Improved	27 (87.1%)
	No changes	4 (12.9%)
	Worsened	0 (0%)
Prosthesis	Teflon wire piston	22 (70.97%)
	Wire piston	9 (29.03%)

Fisch's reversal step stapedotomy was performed in all cases. There was one case of obvious lymphatic leakage as an intraoperative complication. In this case, no sensorineural hearing loss was observed intraoperatively following implantation of the fascia into the oval window and placement of the prosthesis above. In another case, there was frequent and prolonged dizziness during postoperative head positioning, which was thought to be caused by chronic irritation of the otolith by the inserted prosthesis. One year after the initial surgery, the prosthesis was shortened, and the symptoms disappeared. No complications were observed in the other cases.

**Figure 2:**
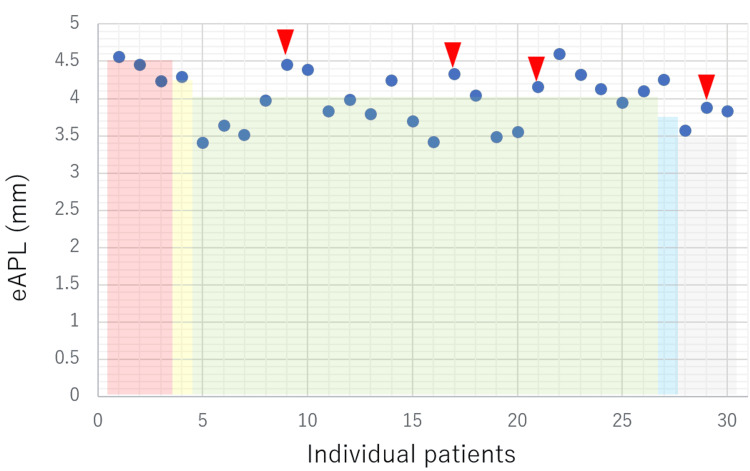
Relationship between the eAPL measured on axial CT images, the inserted prosthesis length, and postoperative hearing outcomes Circles (●) represent individual eAPL values measured on axial CT images for each case. Rectangular frames indicate the length of the actually inserted prosthesis. Triangles (▼) indicate cases with unfavorable postoperative hearing outcomes. Four out of 11 patients (36.4%) showed unfavorable postoperative hearing improvement. eAPL - estimated appropriate prosthesis length

**Figure 3:**
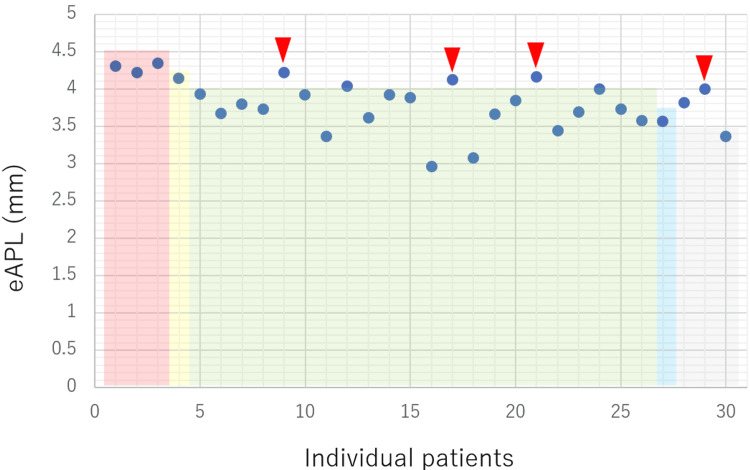
Relationship between the eAPL measured on coronal CT images, the inserted prosthesis length, and postoperative hearing outcomes. Circles (●) represent individual eAPL values measured on coronal CT images for each case. Rectangular frames indicate the length of the actually inserted prosthesis. Triangles (▼) indicate cases with unfavorable postoperative hearing outcomes. Four out of five patients (80.0%) showed unfavorable postoperative hearing improvement. eAPL - estimated appropriate prosthesis length

The relationship between the eAPL measured using preoperative temporal bone CT and the actual length of the surgically inserted piston was examined. The aforementioned cases involving intraoperative lymphatic leakage were excluded because the required prosthesis length was shortened due to the fascia placed in the oval window, but the other 30 cases were examined. This distribution of the eAPL measured on axial and coronal CT images is shown in Figures 2 and 3, respectively. The eAPL was 3.40-4.60 mm (mean, 3.99 mm) in the axial view (Figure 2) and 2.96-4.34 mm (mean, 3.79 mm) in the coronal view (Figure 3). The eAPL tended to be larger in axial view. With respect to the intraoperatively inserted prosthesis length, when the eAPL was measured in the axial view, 15 cases (11 successful and four failed) had eAPL>prosthesis length, and 16 cases (16 successful and zero failed) had eAPL<prosthesis length (Figure 2). In contrast, when the eAPL was measured in the coronal view, five cases had eAPL>prosthesis length (one successful and four failed), and 25 coronal sections had eAPL<prosthesis length (25 successful and zero failed; Figure 3).

## Discussion

Stapes surgery is performed to correct sound transmission disorders caused by poor mobility of the stapes footplate, mainly due to otosclerosis or stapes fixation. In many otologic surgeries, not limited to stapes surgery, microscopic surgery has been standard, with endoscopes used for detailed observation and treatment of the tympanic cavity and blind spots [8]. However, with the improvement of surgical endoscopic video system technology in recent years, TEES, in which all surgical operations are performed transcranially using an endoscope, has gained popularity [9,10]. TEES offers advantages, such as improved visualization around the stapes, reduced canal osteotomy, and less postoperative pain, with hearing outcomes and complication rates comparable to microscopic surgery [11-16]. Nevertheless, TEES has inherent limitations, such as single-handed instrumentation, limited working space, and reduced depth perception compared with microscopic surgery. In addition, difficulty in adequately supporting the long process of the incus during prosthesis insertion has been reported [16]. These factors require substantial surgical experience and may increase procedural complexity. Despite these limitations, when performed by experienced surgeons, TEES allows reliable assessment of stapes anatomy and accurate prosthesis positioning.

In the current study, postoperative hearing outcomes of previous cases using the Japanese Otological Society criteria were evaluated, and 27 successful cases (87.1%) were found, which is comparable to microscopic surgery (79-97.9%) [16-20]. Complications occurred in two cases (intraoperative lymphatic leakage and prolonged postoperative dizziness during head positioning, respectively). The former was handled intraoperatively by placing the fascia in the oval window and then standing the piston up; postoperative sensorineural hearing loss was not observed. The latter was thought to be caused by persistent irritation of the otolith caused by an overly-long prosthesis; following prosthesis replacement two years after the initial surgery, the dizziness symptoms were alleviated. Although the incidence of sensorineural hearing loss after stapes surgery is 5.1-9% [18,21], in the current study, there were no cases of postoperative deterioration of bone conduction hearing.

Poor postoperative hearing after stapes surgery may result from both operator and pathological factors, with improper prosthesis fixation being a major operator-related cause [22]. In the current study, four cases of postoperative conductive hearing loss with persistent air-bone gaps were attributed to prosthesis displacement from poor insertion. Analysis of preoperative temporal bone CT showed that in all cases with poor postoperative hearing improvement, the surgically-inserted prosthesis was shorter than the eAPL, suggesting prosthesis length was a key factor in postoperative hearing. In stapes surgery, a dedicated depth gauge is used intraoperatively to measure the distance between the long process of the incus and the stapes footplate, and a prosthesis of appropriate length is selected. Although preoperative imaging modalities such as CT can provide an estimate of the available piston length, the final prosthesis length is determined by direct intraoperative measurement; therefore, intraoperative measurement remains the current standard practice. Because eAPL is an estimated value derived from preoperative CT imaging, it should not be interpreted as a definitive determinant of prosthesis length. Rather, eAPL is intended to serve as an adjunctive preoperative reference to support surgical planning, while the final selection of prosthesis length must be guided by intraoperative anatomical findings. Furthermore, the prosthesis should be moved after insertion to confirm that it has been inserted appropriately. In all our cases, the mobility of the prosthesis was confirmed after insertion; however, because stereoscopic viewing is not possible during endoscopic surgery, it is sometimes difficult to detect slight misalignment. All 27 successful cases had prostheses longer than the eAPL. In one case, the actual length of the inserted piston was shorter because of intraoperative external lymphatic leakage, which resulted in placement of the fascia on the oval window. The eAPLs measured in the axial view of the temporal bone CT were longer than the prosthesis in successful cases. This is presumably due to the anatomical positioning of the ossicles, which makes it difficult to determine the relationship between the oval window and the long process of the incus on axial-view CT, resulting in a large error. Hence, for eAPL measurement, temporal bone CT in the coronal view is recommended, which allows for a fairly accurate preoperative prediction of the appropriate prosthesis length.

The ability to predict the appropriate preoperative prosthesis length should increase the rate of postoperative hearing improvement. Another advantage of the preoperative prediction of the prosthesis length is that it ensures better control of the supplies used at the time of surgery. In the current study, eAPL was 2.96-4.32 mm, indicating that some cases require a 4.5 mm long prosthesis. Limited stock of pistons may pose challenges if length adjustment or replacement is needed. Although alternative methods, such as autologous cartilage and fascia grafting [23], exist, they are technically demanding and less common. Previous studies investigating the relationship between preoperative CT measurements and prosthesis length are limited [4,5]. One retrospective study demonstrated that high-resolution CT measurements of the distance between the long process of the incus and the stapes footplate correlated significantly with the prosthesis length selected intraoperatively, suggesting that CT may help predict optimal prosthesis length. Another recent study reported strong agreement between preoperative high-resolution CT (HRCT) imaging and intraoperative measurements in estimating the required prosthesis length. However, such reports remain few, and earlier studies have primarily focused on intraoperative measurement techniques or postoperative imaging rather than simple preoperative CT-based predictors. In contrast, the present study used routine coronal CT measurements (eAPL) to evaluate clinical feasibility for predicting prosthesis length. Unlike image analysis software-based reconstructions [24], our method does not use image analysis software and has the advantage of being a simpler method.

The present study has some limitations. First, the sample size was relatively small, and the retrospective study design makes it difficult to determine whether preoperative temporal bone CT evaluation directly contributed to postoperative hearing outcomes. In addition, potential bias related to the type of prosthesis used cannot be excluded.

Second, intra- and inter-rater reliability of the eAPL measurements was not formally assessed. Although all measurements were performed by an experienced examiner using a standardized protocol based on clearly defined anatomical landmarks on routine CT images, minor measurement variability may have influenced the results. Future studies involving multiple independent raters and formal reliability analyses, such as intraclass correlation coefficients, are warranted.

Third, the incudostapedial joint and the oval window are not necessarily located on the same CT plane. Ideally, oblique or three-dimensional CT reconstructions could be used to align these structures on a single plane and measure the true shortest anatomical distance. However, such techniques require dedicated software and additional processing and are not routinely available in daily clinical practice. In the present study, the straight-line distance between these structures was measured on thin-slice coronal and axial CT images as a practical approximation, which was defined as the eAPL. The objective aim was not to determine the exact anatomical shortest distance, but rather to evaluate whether a simple and reproducible CT-based measurement could be clinically useful.

Finally, although three-dimensional CT reconstruction may allow more precise measurements, coronal CT images are universally included in standard temporal bone CT protocols. Therefore, this study intentionally focused on a simple coronal CT-based measurement to assess its feasibility and clinical applicability. Future prospective studies with larger cohorts and direct comparisons among coronal, axial, and three-dimensional CT measurements are needed to further clarify the expected range of measurement error and the clinical utility of this approach.

## Conclusions

Stapes surgery using TEES was reviewed. Postoperative hearing outcomes and complications were comparable to previous reports, indicating that TEES was safe and effective. However, endoscopic limitations, such as a lack of stereoscopic vision, raise concerns about reliably confirming prosthesis placement. Measuring the distance from the oval window to the long process of the incus on preoperative temporal bone CT and estimating eAPL may reduce the risk of prosthesis displacement after surgery. These findings suggest that preoperative CT may enhance surgical planning accuracy and improve intraoperative efficiency during stapes surgery. Such an approach could be especially valuable in training environments and in cases involving complex anatomical variations, leading to better patient outcomes.
